# Lung adenocarcinoma with *EGFR* 19Del and an *ALK* rearrangement benefits from alectinib instead of an *EGFR*-TKI: A case report

**DOI:** 10.1097/MD.0000000000030316

**Published:** 2022-09-02

**Authors:** Hongbiao Wang, Sujuan Zhu, Zhifeng Li, Xiaofang Qi, Liwen Zhang, Leiyu Ke, Yingcheng Lin

**Affiliations:** a Medical Oncology, Cancer Hospital of Shantou University Medical College, Shantou, Guangdong, China; b OrigiMed, Shanghai, China.

**Keywords:** Lung Adenocarcinoma, EGFR/ALK co-mutated, Alectinib

## Abstract

A remarkable concurrence of an *EGFR* mutation and an *EML4-ALK* fusion (double positive) occasionally occurs within a narrow number of patients. Previous studies using targeted therapy on *EGFR/ALK* co-mutated patients have commonly focused on single tyrosine kinase inhibitors (TKIs) or on the sequential use of *EGFR*-TKIs and *ALK*-TKIs. At present, no consensus exists regarding the treatment of patients with double positive mutations. The effectiveness of precision therapy also remains unknown.

**Patient concerns:**

A 53-year-old female non-smoker who described recurrent coughing and blood in her sputum over a month-long interval was examined at a local hospital.

**Diagnosis:**

Using computed tomography (CT) and positron emission tomography CT (PET-CT), the patient was diagnosed with Stage IVb lung adenocarcinoma (T4N3M1).

**Interventions:**

The patient had a novel *ALK-RAB10* rearrangement identified using DNA sequencing, which, at the transcript level, was actually a canonical *ALK* fusion that caused a response to alectinib therapy.

**Outcomes:**

The patient has achieved partial remission (PR), with a progression free survival (PFS) of 16 months, and continues to benefit.

**Lessons:**

Our results may indicate differential sensitivities to TKIs in patients harboring an *EGFR* mutation and an *ALK* rearrangement. Our patient’s response to alectinib, instead of to *EGFR*-TKIs, may lead to an expanded list of alectinib beneficiaries who have rare gene co-alterations in lung adenocarcinoma.

## 1. Introduction

Over the past few years, multiple genomic breakpoints in fusion variants have been detected using DNA sequencing. Li et al^[1]^ determined rare fusion variants identified using DNA-based next generation sequencing (NGS) that did not generate expressed fusion or canonical fusion transcripts. The result suggests the potential unreliability of genomic breakpoint locations in predicting biological outcomes. As such, to guide optimal treatment, additional validation using RNA-based NGS, immunohistochemistry (IHC), and fluorescence in situ hybridization (FISH) should be performed in uncommon genomic breakpoint fusion cases.

The coexistence of an *EGFR* mutation and an *EML4-ALK* rearrangement (double positive) has occasionally been determined in a narrow number of patients.^[2–4]^ Currently, no unified opinion exists regarding therapeutics for patients with double positive alterations. Studies have shown that during the prealectinib era, *EGFR* inhibition was an integral part of treatment for patients with *EGFR/ALK* co-alterations, whereas *ALK* inhibition via crizotinib has been shown not to display a more dramatic therapeutic effect.^[5–7]^

Here, we report a case concurrently harboring an *EGFR* 19Del mutation and an *ALK* fusion (a novel *ALK-RAB10* rearrangement identified using DNA sequencing that is actually a canonical *ALK* fusion at the transcript level), which yielded a positive response to alectinib therapy. Based on the effectiveness achieved by alectinib, increasing evidence supports the notion that alectinib, as an *ALK*-TKI, may be more effective than *EGFR*-TKIs in cases displaying the coexistence of an *EGFR* mutation and an *ALK* rearrangement (double positive).

## 2. Case presentation

**Figure 1.**
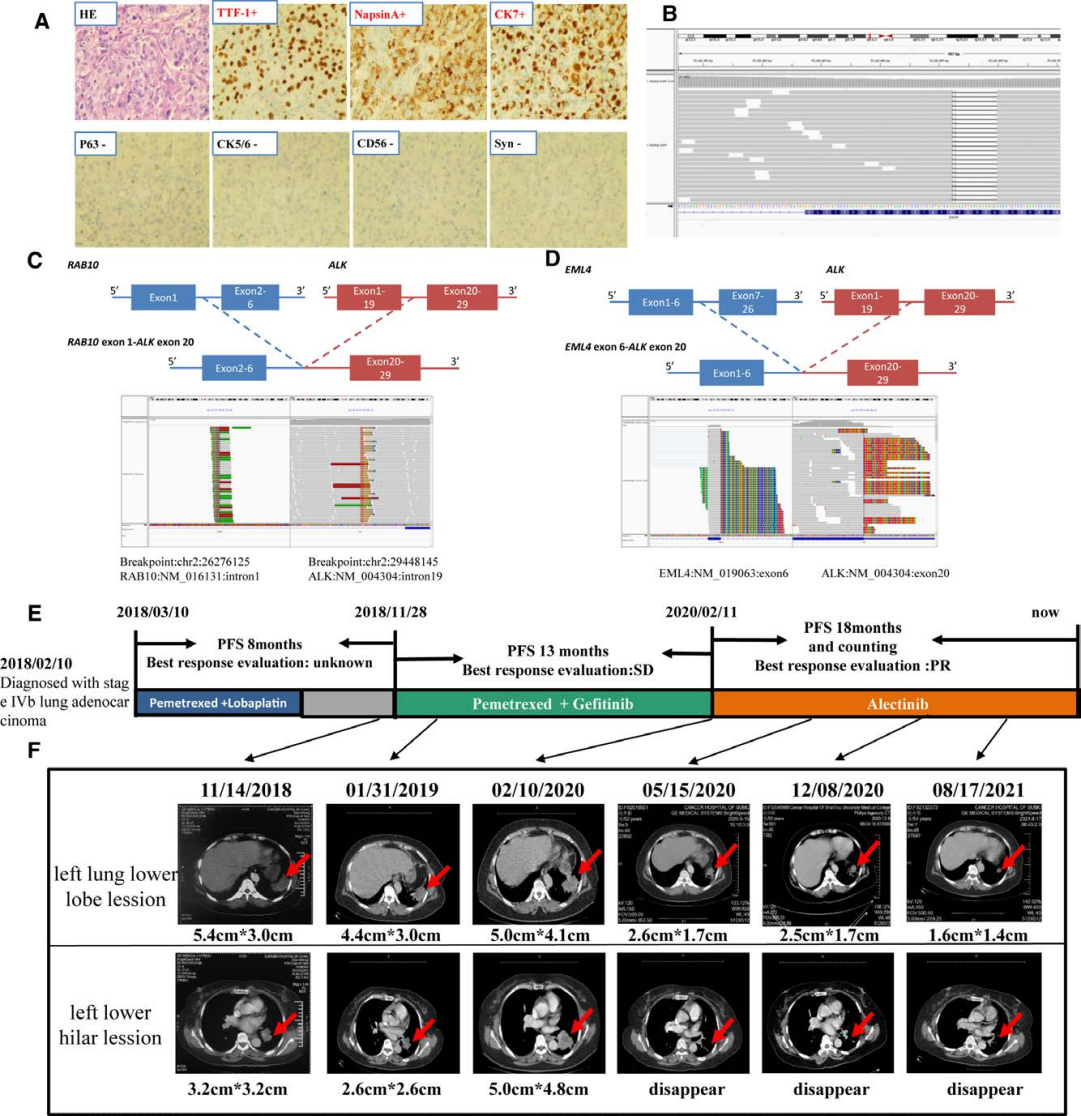
An *EGFR* and *ALK* fusion co-mutation in a patient with lung adenocarcinoma, as well as dynamic monitoring of the treatment response. (A) Hematoxylin and eosin staining and IHC revealed lung adenocarcinoma (200x). (B) Sequencing reads for *EGFR* were viewed via the Integrative Genomics Viewer. (C-D) A schematic of the intergenic region of the fusion and sequencing reads for *ALK* at the DNA and RNA level, respectively. (E) The various treatments the patient received, as well as the duration of each treatment. (F) CT scans revealed lesions within the left lower lobe of the lung and the left lower hilar following subsequent lines of treatment.

In February 2018, a 53-year-old, female nonsmoker who described recurrent coughing and blood in her sputum over a month-long interval was examined at a local hospital. Using computed tomography (CT) and positron emission tomography CT (PET-CT), the patient was diagnosed with Stage IVb lung adenocarcinoma (T4N3M1). Two lesions (8.9 cm × 5.3 cm × 4.2 cm and 6.0 cm × 3.3 cm × 4.4 cm) were detected within the left lung, in addition to multiple lymph node and brain (1.5 cm × 1.4 cm) metastases (images not provided). The patient received 6 cycles of domestic pemetrexed (0.8 g) plus intravenous lobaplatin (50 mg) every 3 weeks from March to August 2018. The patient was then transferred to our hospital for further diagnosis and treatment. In November 2018, a chest CT and brain magnetic resonance imaging (MRI) revealed a 5.4 cm × 3.0 cm mass within the left lower lobe of the lung, a 3.2 cm × 3.2 cm mass within the left lower hilar, a 0.8 cm × 0.9 cm mass within the left parieto-occipital junction area, and mediastinal lymph node metastasis. Based on results from hematoxylin and eosin staining, as well as IHC (Fig. 1A), the pathological diagnosis was again confirmed as lung adenocarcinoma (T4N3M1, Stage IV).

A needle biopsy specimen was additionally sent to OrigiMed (Shanghai, China) for NGS analysis.

The NGS analysis revealed a novel *ALK-RAB10* rearrangement and an exon 19 deletion of *EGFR* at the DNA level (Fig. 1B,C). To validate the location of the breakpoint, as well as the fusion partner at the transcript level, RNA-based NGS was performed. Interestingly, the actual fusion partner of the patient was *EML4-ALK* (Fig. 1D). Given the exon 19 deletion for *EGFR*, the patient began receiving 16 cycles of domestic pemetrexed (0.9 g) plus intravenous gefitinib (250 mg) every 3 weeks from November 2018 to February 2020 (Fig. 1E).

For the first 6 weeks of treatment, disease remained stable. However, following 16 cycles of Pem plus G treatment, the lesion in the lower lobe of left lung and lesion in the left lower hilar began to increase in size (5.2 cm × 3.5 cm and 5.0 cm × 4.8 cm, respectively), indicating disease progression. Due to the *EML4-ALK* fusion, in February 2020, the patient began alectinib treatment (600 mg, twice per day). Since April 2021, the patient has achieved partial remission (PR), with a progression free survival (PFS) of 16 months, and continues to benefit (Fig. 1E and F).

## 3. Discussion

*EGFR/ALK* co-alterations and the *ALK* rearrangement of an uncommon genomic breakpoint were determined as the actual canonical fusion. Both occurrences are very rare in lung adenocarcinoma. Procedures for treating patients with concomitant *EGFR/ALK* co-alterations are sometimes controversial. Zhou et al^[6]^ first reported that a lung adenocarcinoma patient with an *EGFR*-mutant and an *ALK* fusion acquired resistance to osimertinib, but was sensitive to the combined treatment of gefitinib plus crizotinib. Schmid et al^[8]^ stated that *EGFR*-TKIs may display better outcomes, as compared to *ALK*-TKIs, in patients with an *EGFR* mutation and an *ALK* rearrangement. However, Won et al^[9]^ reported the opposite and stated that *ALK*-TKIs may be preferred in patients with concomitant *EGFR/ALK* alterations. Qin et al^[5]^ recently reported that a female lung adenocarcinoma patient with brain metastases and the coexistence of an *EGFR* mutation/*DCTN1-ALK* translocation responded well to successive osimertinib and alectinib treatment. These limited studies may indicate that patients harboring both an *EGFR* mutation and an *ALK* rearrangement exhibit different sensitivities to both types of TKI therapies, suggesting different degrees of dependence on *EGFR* and *ALK* oncogenes.^[10,11]^

Our interest in this case arose from the fact that this case is the first report of a durable, dramatic response to alectinib in a lung adenocarcinoma patient harboring both an *EGFR* mutation and an *ALK* rearrangement for the uncommon genomic breakpoint. For a co-altered patient, the total PFS achieved using an *ALK*-TKI treatment was longer than the PFS achieved when using an *EGFR*-TKI treatment. A nearly complete response was also achieved with alectinib (a PFS of 13 months vs a PFS of 18 months). Such a discrepancy may indicate that the proportion of *ALK* amplification, as an oncogene driver, is higher than that of an *EGFR* mutation, or that the application of alectinib is more effective than *EGFR*-TKIs in the treatment of co-mutated lung adenocarcinoma patients.

Although a recent study also reported that alectinib was an effective treatment in patients with a co-mutation,^[5]^ at present, we are still unable to draw definite conclusions. Our study suggests that additional validation using RNA-based NGS, IHC, or FISH should be performed for uncommon genomic breakpoint fusion. Based on our results to date, a subgroup of patients with a coexisting *EGFR* mutation and an *ALK* fusion may benefit from the treatment outlined.

## 4. Conclusion

The case presented provides a new reference for understanding *ALK* fusion mutations. Using DNA sequencing, we identified a novel *ALK-RAB10* rearrangement within a lung adenocarcinoma patient that is actually a canonical *ALK* fusion at the transcript level. We also discussed the possibility for the future application of alectinib as an *ALK*-TKI for lung adenocarcinoma patients having an *EGFR* mutation and an *ALK* rearrangement (double positive). Despite our findings, at this stage, drawing clear conclusions regarding clinical characteristics and patient treatment outcomes is difficult. Long-term follow-up for the patient described in our study is ongoing, and we will continue to pay close attention as to whether alectinib resistance develops over time.

## Acknowledgments

We thank the patient and her family. Informed consent for publication was obtained from the patient and her family.

## Author contributions

Conceptualization: Yingcheng Lin, Sujuan Zhu

Data curation: Hongbiao Wang, Zhifeng Li, Xiaofang Qi, Liwen Zhang, Leiyu Ke

Writing—original draft: Hongbiao Wang

Writing—review & editing: Zhifeng Li, Yingcheng Lin
